# *Acanthamoeba castellanii* Can Facilitate Plasmid Transfer Between Environmental *Pseudomonas* spp

**DOI:** 10.1002/jobm.70051

**Published:** 2025-05-11

**Authors:** Maarten J. Sarink, Lara Grassi, Aloysius G. M. Tielens, Annelies Verbon, Margreet C. Vos, Wil Goessens, Nikolaos Strepis, Corné H. W. Klaassen, Jaap J. van Hellemond

**Affiliations:** ^1^ Department of Medical Microbiology and Infectious Diseases, Erasmus MC University Medical Center Rotterdam Rotterdam the Netherlands; ^2^ Department Internal Medicine University Medical Center Utrecht Utrecht the Netherlands

**Keywords:** *Acanthamoeba*, antimicrobial resistance, horizontal gene transfer, plasmids, *Pseudomonas aeruginosa*

## Abstract

The conditions in which antimicrobial resistance (AMR) genes are transferred in natural environments are poorly understood. *Acanthamoeba castellanii* (a cosmopolitan environmental amoeba) feeds on bacteria by phagocytosis, which places the consumed bacteria closely together in a food vacuole (phagosome) of the amoeba. This way, amoebae can facilitate genetic exchanges between intra‐amoebal bacteria. We studied this phenomenon in the clinically relevant bacteria *Pseudomonas oleovorans* and *Pseudomonas aeruginosa* (strain 957). The internalization of both the plasmid donor and recipient bacteria was shown by confocal microscopy. In seven independent experiments, an on average 12‐fold increase in transfer of the *bla*_VIM‐2_ gene between these two *Pseudomonas* strains was observed in the presence of *A. castellanii* compared to its absence. Negligible or no plasmid transfer was observed from *P. oleovorans* to 18 other investigated strains of *P. aeruginosa*. AMR gene transfer via plasmids between *Pseudomonas* species is highly strain‐dependent and *A. castellanii* can substantially enhance plasmid transfer. This process of plasmid transfer might also occur between other bacteria and predatory protozoa, such as amoebae that reside in the gut of humans and animals.

**Abbreviations**
AMR: antimicrobial resistance
CFU: colony‐forming unit
CRPA: carbapenem‐resistant *Pseudomonas aeruginosa*
DMSO: dimethyl sulfoxide
FITC: fluorescein isothiocyanate
HGT: horizontal gene transfer
MALDI‐TOF‐MS: matrix‐assisted laser desorption/ionization time‐of‐flight mass spectrometry
MH: Mueller Hinton
PBS: phosphate‐buffered saline
PYG medium: peptone‐yeast‐glucose medium
TSA: trypticase soy agar

## 1 Introduction

Antimicrobial resistance (AMR) has been declared by the World Health Organization to be one of the top 10 global public‐health threats facing humanity [1]. Misuse and overuse of antibiotics are the main drivers for selection of bacteria that are resistant to antibiotics. In addition, AMR can spread via horizontal gene transfer (HGT) of AMR genes. The conditions in which AMR genes are transferred in natural environments are poorly understood, although stress, nutrient supply and cell density can affect the HGT rate [2]. Studies in natural aquatic environments revealed that biofilms are so‐called hotspots for HGT [2]. Biofilms are composed of a mixture of organisms, containing prokaryotes as well as eukaryotes. *Acanthamoeba* spp. are eukaryotes often present in biofilms. This unicellular protozoa feeds on bacteria by phagocytosis, and in this process places bacteria together in a food vacuole (phagosome). Most bacteria are lysed and degraded, but some can survive or even kill the amoeba [3, 4, 5]. One of the bacterial species that can survive predation by *Acanthamoeba* spp. is *Pseudomonas aeruginosa*, which can live and multiply in the food vacuoles of this amoeba [6]. *P. aeruginosa* and *Acanthamoeba* spp. were shown to be present together in floor drains of a general hospital [7]. *P. aeruginosa* can cause opportunistic nosocomial infections in humans that are often hard to treat, due to reduced susceptibility to multiple antibiotics. Resistance to the last‐line antibiotic class of carbapenems is especially worrisome, as bloodstream infections with a carbapenem‐resistant *P. aeruginosa* (CRPA) result in increased mortality compared to infections with a carbapenem‐susceptible counterpart [8]. The Verona‐Integron‐encoded‐metallo‐β‐lactamase (*bla*_VIM‐2_) resistance gene, which results in CRPA, is not only found in *P. aeruginosa*, but also in nonpathogenic *Pseudomonas* spp., which could act as a reservoir of AMR genes [9]. Amoebae can act as a place that favors genetic exchanges between bacteria [5, 10]. In the food vacuoles (phagosomes) of amoebae, different bacteria from the environment will end up together in closed vacuoles, which might enhance the transfer of mobile genetic elements (e.g., plasmids). Hence, amoebal predation could facilitate HGT of AMR genes [5]. This concept is rarely studied with clinically relevant bacteria. Therefore, we examined the transfer rate of a plasmid encoding the *bla*_VIM‐2_ gene, between two different *Pseudomonas* species (*P. oleovorans* to *P. aeruginosa*) in the presence and absence of *A. castellanii*.

## 2 Materials and Methods

### 2.1 Strains

*Acanthamoeba castellanii* ATCC strain 30010 was grown in cell culture flasks at 25°C in PYG medium, which contains proteose peptone, yeast extract and glucose with salt additives (ATCC medium 712) [11]. This medium was supplemented with 40 µg/mL gentamicin, 100 units/mL penicillin and 100 µg/mL streptomycin. The *Pseudomonas oleovorans* donor strain carrying a plasmid harboring the *bla*_VIM‐2_ gene was isolated from a hospital sink in the Erasmus MC University Medical Center Rotterdam, the Netherlands. All used *Pseudomonas aeruginosa* recipient strains were isolated from wet hospital environments in the Erasmus MC, except for *P. aeruginosa* ATCC strain 27853, which was obtained from the American Type Culture Collection (Manassas, Virginia, USA). Bacteria were grown on Trypticase Soy Agar (TSA) II with 5% Sheep Blood (BD Biosciences, Franklin Lakes, New Jersey, USA).

### 2.2 Fluorescent Labeling and Confocal Imaging

The intracellular location of both *Pseudomonas* species after phagocytosis by *Acanthamoeba* was examined by fluorescent labeling and confocal microscopy. *P. oleovorans* was fluorescently stained using 1 mg/mL fluorescein isothiocyanate isomer I (FITC) (Sigma‐Aldrich, USA) and *P. aeruginosa* strain 957 was separately stained using 1 mg/mL lectin PNA, Alexa Fluor 647 Conjugate (Invitrogen, USA) suspended in DMSO (MERCK KGaA, Germany). Staining of both *Pseudomonas* species was performed by incubation of the bacteria with the dye in PBS with 5% glycerol (Sharlau, Spain) and 2 mM MgCl_2_ (Sigma‐Aldrich, USA) for 1 h, while gently swirling, at room temperature. Subsequently, the excess dye was removed by washing with PBS 5 and 3 times, respectively. *A. castellanii* were then incubated in PBS for 1 h at 25°C with both stained *Pseudomonas* species in the ratio of 1:1:1. After incubation, the samples were fixated for 30 min on ice in 8% formaldehyde (MERCK KGaA, Germany) before imaging. Confocal images were taken using the Leica SP5 confocal laser scanning microscope (Leica, Germany), deconvoluted with the Huygens software (SVI, Hilversum, The Netherlands) and reconstructed using Fiji37.

### 2.3 Gene Transfer Experiments

To harvest *A. castellanii* trophozoites, cell culture flasks were placed on ice for 20 min, after which the nutrient‐rich medium was removed by two subsequent washing steps with 1% PYG in PBS (v/v). *P. oleovorans* and *P. aeruginosa* strains were grown overnight on TSA, after which an 0.5 optical density (OD) at 600 nm suspension was prepared in 1% PYG in PBS. A predetermined quantity containing 1 × 107 *P. oleovorans* and 1 × 107 of one of the *P. aeruginosa* strains were added to each well of a 24‐well plate with or without 2 × 105 *A. castellanii* in 1% PYG in PBS in a total volume of 1 mL. The applied in vitro co‐culture conditions (1% PYG in PBS without shaking) were a mimic of the low nutrient situation in the hospital water system [12]. After incubation for 24 h at 25°C the 24‐well plate was placed on ice for 20 min. Next, the content of each well was collected by vigorous pipetting and transferred into tubes containing 250–280 mg of 1 mm diameter glass beads. To release viable intracellular bacteria from the amoeba, bead‐beating was performed 16 times for 30 s on and 30 s off. This bead‐beating procedure did not affect the viability of the *Pseudomonas* spp. (Figure S1). After bead‐beating, serial dilutions were plated on TSA as well as on Mueller Hinton (MH) II agar plates containing 4 mg/L meropenem, 2 mg/L tobramycin and 25 mg/L 1,10‐phenanthroline. Resistance genes encoding for meropenem and tobramycin‐resistance are plasmid encoded and therefore meropenem and tobramycin were applied as selective agents. To select for *P. aeruginosa*, 1,10‐phenantroline was used, as described in [13]. TSA plates were incubated for 24 h at 37°C, after which colonies of *P. oleovorans* and *P. aeruginosa* could be quantified, as colonies of both species could be differentiated by their different morphology on TSA agar plates. MH plates with selective compounds were incubated for 48 h at 37°C. Plasmid transfer rate was determined by dividing the CFU on selective plates by the total number of CFU (donor + recipient expressed in 10^8^) present at the end of the incubation. All experiments were performed in duplicate or more where indicated.

### 2.4 Real‐Time PCR, MALDI‐TOF and Conventional PCR

In each gene‐transfer experiment for each condition, three colonies were examined by both real‐time PCR and Matrix‐assisted laser desorption/ionization with time‐of‐flight mass spectrometry (MALDI‐TOF‐MS) (Bruker, Billerica, Massachusetts, USA) to confirm the presence of the *bla*_VIM‐2_ gene and the *P. aeruginosa* identity, respectively. Real‐time PCR was performed in a LightCycler 96‐well plate (Roche, Basel, Switzerland) by mixing a small aliquot of a colony with 10 μL of LightCycler 480 Probes Master (Roche), 5 μL of *bla*_VIM_‐PCR probe mix (Table S1) and 5 μL of PCR‐grade water [14]. Samples were run in a LightCycler 480II instrument (Roche) with pre‐denaturation at 95°C for 5 min; amplification of 50 cycles between 95°C for 5 s and 60°C for 30 s and final cooling at 40°C for 1 min.

Conventional PCR was used to examine the integrity of the *bla*_VIM‐2_ containing plasmid after incorporation of the *bla*_VIM‐2_ gene in the recipient *P. aeruginosa*−957 strain. Primers were designed for six regions in the original plasmid in *P. oleovorans*, located 20–30 kb from each other (Table S1). All primers were used at a concentration of 0.5 μM. In a 96‐well plate (Roche), 5 μL of extracted DNA was mixed with 1.25 μL of forward primer, 1.25 μL of reverse primer, 12.5 μL of PCR Faststart PCR Master Mix (Roche) and 5 μL of PCR‐grade water. Samples were run in a Biometra TAdvanced PCR thermocycler instrument (Labgene Scientific SA, Châtel‐St‐Denis, Switzerland) with the following conditions: pre‐denaturation at 95°C for 10 min; 30 cycles of denaturation at 95°C for 30 s, annealing at 55°C for 30 s, and extension at 72°C for 1 min; followed by final extension for 10 min at 72°C and cooling to 4°C. The DNA amplicons were separated by electrophoresis on a 2% agarose gel (Sphaero Q, Gorinchem, The Netherlands, and Thermo Scientific, Waltham Massachusetts, USA) and visualized by staining with SYBR Safe DNA Gel Stain (Invitrogen, Carlsbad, California, USA).

## 3 Results

### 3.1 Ingestion of *Pseudomonas* by *A. castellanii*

**Figure 1:**
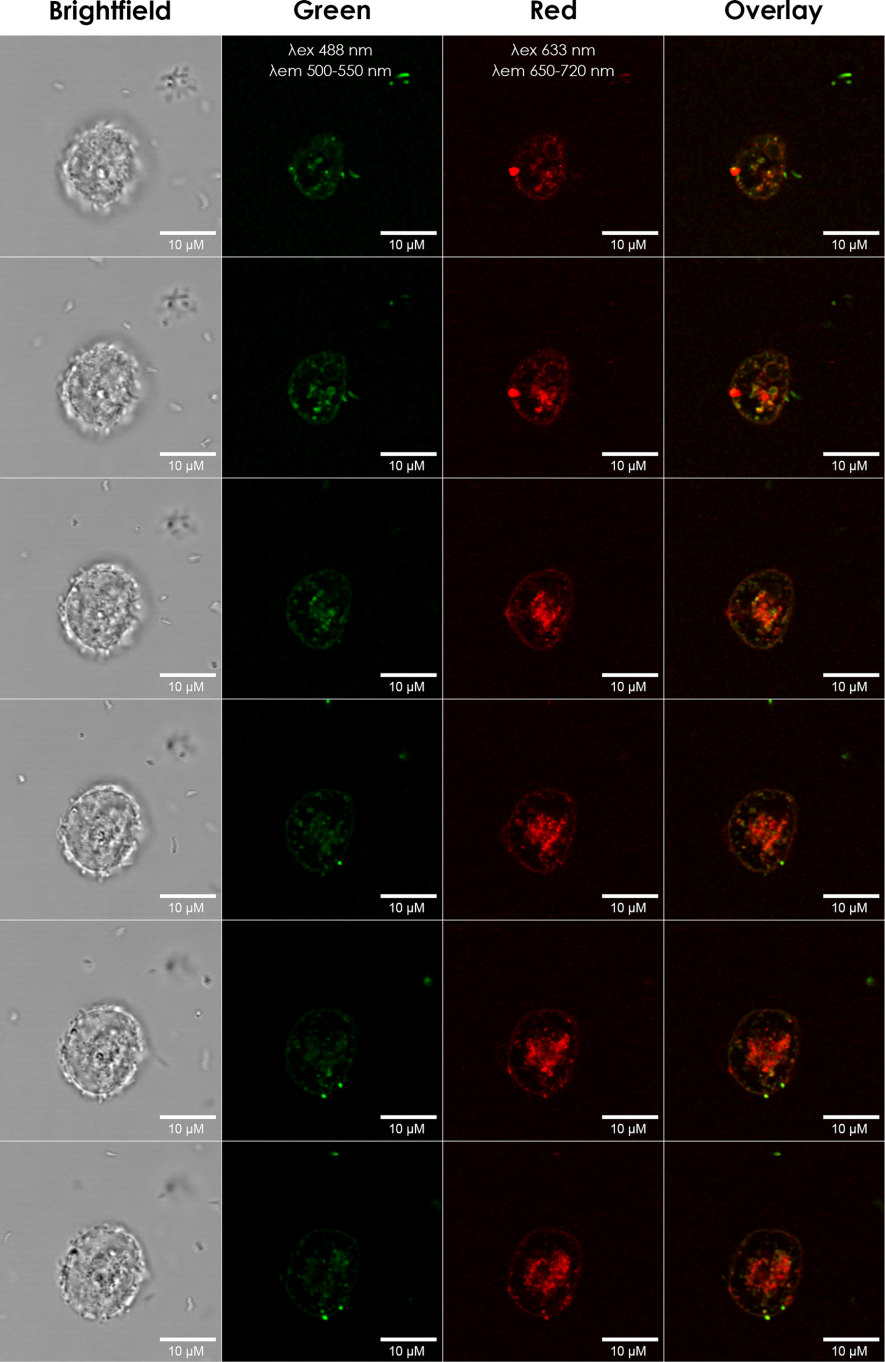
Images taken by confocal microscopy of co‐cultures with *A. castellanii*, *P. oleovorans* and *P. aeruginosa*−957. Shown are z‐stacks (sections of 1 µm) of bright field and fluorescent images at indicated wavelengths. *A. castellanii* was incubated together with *P. oleovorans* (stained by FITC—shown in green) and *P. aeruginosa*−957 (stained with lectin PNA bound to Alexa 647—shown in red) in PBS for 1 h, after which samples were fixated and examined by confocal microscopy. The scale bars are 10 µm.

**Figure 2:**
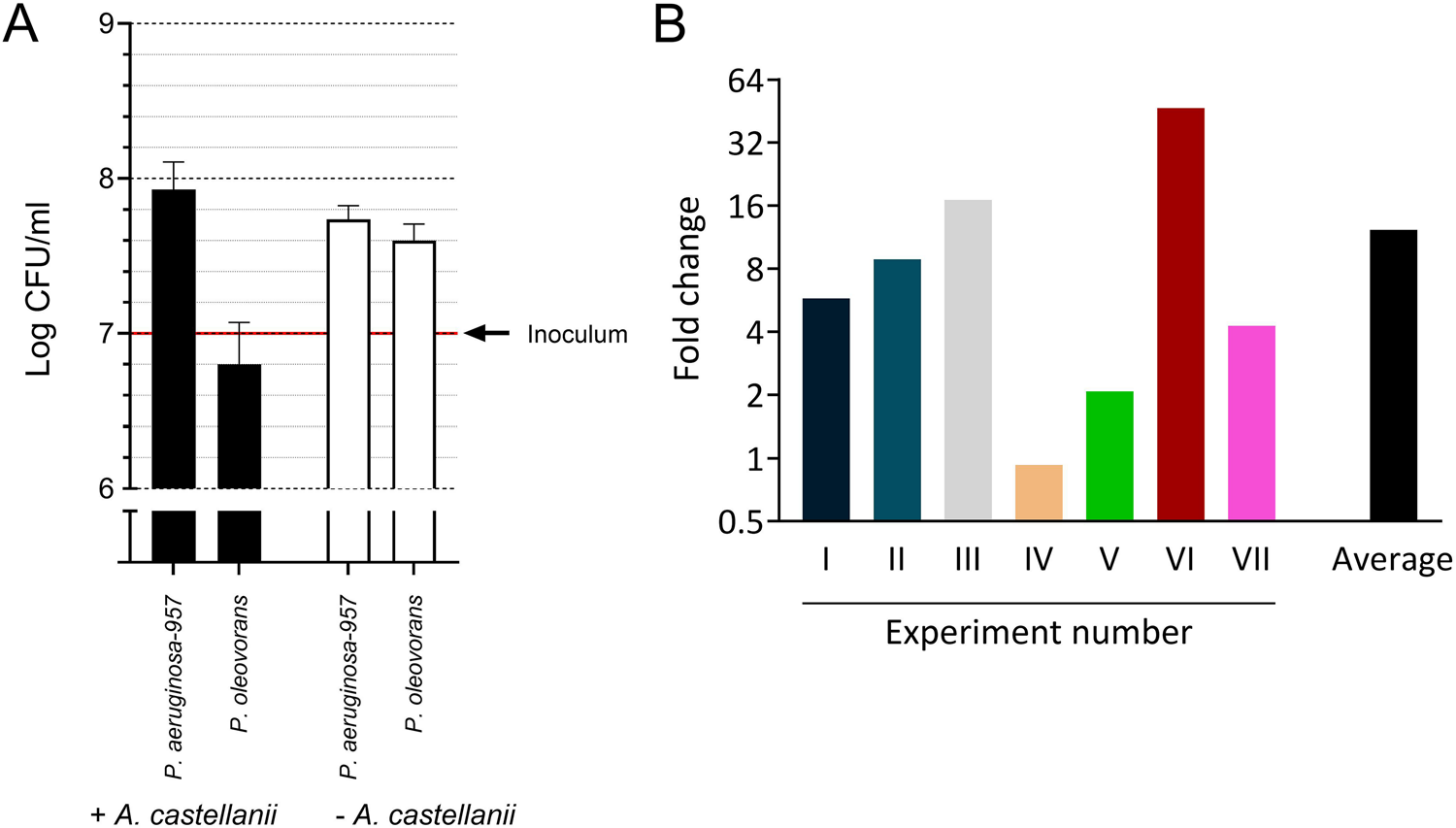
Transfer rate of the plasmid encoded *bla*_VIM‐2_ gene from *Pseudomonas oleovorans* to *Pseudomonas aeruginosa*−957 in the presence or absence of *Acanthamoeba castellanii*. (A) shows the number of colony‐forming units (CFU) on tryptic soy agar of *P. oleovorans* and *P. aeruginosa*−957 after 24 h of incubation in a low‐nutrient medium (1% PYG in PBS (v/v)) in the presence (+*A. castellanii*) or absence (−*A. castellanii*) of *A. castellanii*. 1 × 107 *P. oleovorans* and 1 × 107 *P. aeruginosa*−957 (indicated with a red line) were added at the start of the incubation, with or without 2 × 105 *A. castellanii*. (B) shows the difference in transfer rate in fold change in the recipients/10^8^ CFU formed by *P. oleovorans* and *P. aeruginosa*−957 in the presence of *A. castellanii* compared to its absence. Culture conditions were used as described for (A). After 24 h of co‐culture, the contents were added to tryptic soy agar to determine CFU counts as well as to selective plates containing 4 mg/L meropenem, 2 mg/L tobramycin and 25 mg/L 1,10‐phenanthroline to determine the number of recipients.

The uptake by *A. castellanii* of both *P. aeruginosa*−957 and *P. oleovorans* after 90 min of co‐incubation was shown by confocal microscopy, as the distinctly fluorescent‐stained *Pseudomonas* species were both observed inside *A. castellanii* (Figure 1). This result confirms our previous study which showed the intracellular presence of *P. aeruginosa* in *A. castellanii* [3]. Subsequently, the growth dynamics of both *Pseudomonas* species were examined in the presence and absence of *A. castellanii* in a low‐nutrient medium. This demonstrated that of both *Pseudomonas* species the total number increased in the absence of *A. castellanii* (Figure 2A). In the presence of *A. castellanii*, the number of *P. oleovorans* dropped substantially whereas the number of *P. aeruginosa*−957 increased. It is known that bacterial evolution resulted in bacteria resistant to destruction by free‐living amoebae [4, 5, 6]. This could explain why *A. castellanii* trophozoites did not digest the *P. aeruginosa*‐957 strain.

### 3.2 Determination of the Transfer Efficiency

The transfer efficiency of the *bla*_VIM‐2_ gene from *P. oleovorans* to the *P. aeruginosa*−957 strain was examined by co‐culturing these two *Pseudomonas* species together for 24 h in the presence or absence of *A. castellanii*, followed by examination of the number of bacteria on selective as well as nonselective agar plates. Real‐time PCR and MALDI‐TOF analysis were performed to confirm the presence of the *bla*_VIM‐2_ gene and *P. aeruginosa* identity of the obtained colonies on the selective agar plates, respectively. In total, this experiment was performed seven times. It demonstrated an on average 12‐fold (range 1–47) higher plasmid transfer rate in the presence of *A. castellanii* compared to its absence (Figure 2B). The plasmid‐transfer rate per 10^8^ bacteria ranged from 2000 to 224,625 in the absence of *A. castellanii* and from 11,530 to 1,990,196 in the presence of *A. castellanii*. The presence of the entire plasmid (size: 158 kbp) with the *bla*_VIM‐2_ gene in colonies growing on the selective plates was confirmed by additional conventional PCR analysis (Figures S2–S3).

This study started with an experiment where the plasmid‐transfer efficiency was tested twice with in total 19 *P. aeruginosa* strains: ATCC strain 27853 plus 18 strains isolated from wet hospital environments, including strain 957 described above. The experiment showed that the *bla*_VIM‐2_ gene could be transferred from *P. oleovorans* to *P. aeruginosa* in the above‐mentioned strain 957, and in only three other *P. aeruginosa* strains, but in these three strains the plasmid transfer rate was negligible compared to the rate with *P. aeruginosa*−957 (Table S2). Furthermore, the transfer rate in these three strains was not substantially increased in the presence of *A. castellanii*.

## 4 Discussion

*Acanthamoeba* spp. perform a dual function as environmental predators that greedily consume other microbes, but act also as hosts for a range of microorganisms that resist phagocytic digestion by the amoebae. Due to the random feeding feature of *Acanthamoeba*, the intracellular multimicrobial communities in the same food vacuole can serve as a “genetic melting pot” [15]. Survival of phagocytosed bacteria and binary fission of bacterial cells inside vacuoles has been described, suggesting ongoing symbiotic or transient interactions, with some bacteria being retained while others are digested [16, 17]. In a study on the intracellular microbiome composition of bacterivorous *Acanthamoeba* isolates, undigested as well as digested bacteria were observed within the same phagocytic vacuole [17]. Our experiments indicate that the *P. oleovorans*, the plasmid donor, was digested, while the recipient, *P. aeruginosa*−957, was resistant to digestion by *A. castellanii*. Digestion of the donor does not necessarily prevent transfer of plasmid. The principal mechanism of plasmid transfer among bacteria is conjugation, which requires cell‐cell contact involving pilus formation for the direct transfer of DNA from the cytoplasm of one bacterial cell to another. However, another possibility is plasmid transformation, which is the uptake of free plasmid DNA, and it has been shown that *P. aeruginosa* is capable of natural transformation [18, 19]. Our experiments showed that AMR gene transfer between *Pseudomonas* species is highly strain‐dependent. This indicates that there are specific strain properties that determine the rate of acquisition, which could be due to the plasmid incompatibility groups of the examined strains [20], although many other factors could be of influence [21].

The presence of a large variety of AMR genes in *Acanthamoeba* spp. present in wastewater has been reported, which suggests that the bacteria inside amoebae may significantly contribute to the transmission of these genes in the surrounding environments [22]. Since the investigated *Pseudomonas* strains in this study were isolated from wet hospital environments, our observation is clinically relevant because transfer of AMR genes between bacteria can occur in hospital‐water systems after which resistant bacteria can spread within the hospital and to the community [23]. The enhanced AMR transfer by *A. castellanii* could also apply to other predatory protozoa, such as amoebae that reside in the gut of humans and animals. The gastrointestinal tract plays an important role in the transfer of plasmids and can host many different protozoa [24]. In this way, protozoa could also have a big impact on AMR transfer among bacteria in the gastrointestinal tract environment of humans.

## Author Contributions

**Maarten J. Sarink:** conceptualization, data curation, formal analysis, visualization, writing – original draft, methodology, writing – review and editing. **Lara Grassi:** investigation, writing – review and editing. **Aloysius G. M. Tielens:** conceptualization, supervision, writing – review and editing. **Annelies Verbon:** funding acquisition, writing – review and editing, supervision. **Margreet C. Vos:** writing – review and editing, resources. **Wil Goessens:** writing – review and editing, methodology. **Nikolaos Strepis:** methodology, investigation, writing – review and editing. **Corné H. W. Klaassen:** methodology, writing – review and editing, investigation. **Jaap J. van Hellemond:** conceptualization, supervision, writing – review and editing, funding acquisition, resources.

## Conflicts of Interest

The authors declare no conflicts of interest.

## Supporting information

065SupportingInformation.

## Data Availability

The data that supports the findings of this study are available in the Supporting Information of this article.
