# Supplementary material for: Acanthamoeba castellanii Can Facilitate Plasmid Transfer Between Environmental Pseudomonas spp
Source: J Basic Microbiol. 2025 May 11;65(8):e70051. doi: 10.1002/jobm.70051 (PMC12319522; doi:10.1002/jobm.70051)
Supplement: Supplementary file 1 — 065SupportingInformation. [file JOBM-65-e70051-s001.pdf]

# Supporting information

## Tables S1 – S2 and Figures S1 – S3

## Table S1. Primer sequences of target genes.

| **Target gene** | **Primer** | **Sequence** |
|---|---|---|
| *bla*$_{VIM-2}$ | Forward | GCAAATTGGACTTCCYGTAA |
| | Reverse 1 | GACGGTGATGCGTACGTTG |
| | Reverse 2 | CCCTAAGGGCATCAACTCC |
| *dotG* | Forward | GGCATCTTCAATCACGGTTT |
| | Reverse | GGGGCTGGTTATTGGTTTTT |
| *pdeF* | Forward | GTGCTGTCTCGCATTACGAA |
| | Reverse | CTCTATCGTCAGCACCGACA |
| *chpB* | Forward | CCATTCCTATCACCGAGCAC |
| | Reverse | GCAGGGCTTCATCTATGGTC |
| *bla* | Forward | GCAAATTGGACTTCCTGTAA |
| | Reverse | CGCTCGATGAGAGTCCTTCT |
| *mobA* | Forward | GTCTCCATCGCCTTCACCT |
| | Reverse | GCACACATCCTGTTCACCAC |
| *pilP* | Forward | TAATCCCGGAGGAATACACG |
| | Reverse | CCAAGCAGGAAGAAGAGCTG |

**Table S2. Efficiency of transfer of a plasmid encoding *bla*$_{VIM-2}$ from *P. oleovorans* to different *P. aeruginosa* strains.**

| Recipient strain | Recipients/10$^8$ bacteria a) | | | |
|---|---|---|---|---|
| | 1st experiment | | 2nd experiment | |
| | with *A. castellanii* | without *A. castellanii* | with *A. castellanii* | without *A. castellanii* |
| *P. aeruginosa* 559 | 0 | 0 | 0 | 0 |
| *P. aeruginosa* 620 | 0 | 0 | 0 | 0 |
| *P. aeruginosa* 623 | 1 | 0 | 16 | 10 |
| *P. aeruginosa* 630 | 0 | 0 | 0 | 0 |
| *P. aeruginosa* 631 | 28 | 19 | 12 | 14 |
| *P. aeruginosa* 772 | 0 | 0 | 0 | 0 |
| *P. aeruginosa* 781 | 0 | 0 | 0 | 0 |
| *P. aeruginosa* 957 | 11530 | 2000 | 1990196 | 224625 |
| *P. aeruginosa* 959 | 0 | 0 | 0 | 0 |
| *P. aeruginosa* 960 | 0 | 0 | 0 | 0 |
| *P. aeruginosa* 1201 | 0 | 0 | 0 | 0 |
| *P. aeruginosa* 1203 | 0 | 0 | 0 | 0 |
| *P. aeruginosa* 1212 | 0 | 0 | 0 | 0 |
| *P. aeruginosa* 1383 | 2 | 0 | 2 | 2 |
| *P. aeruginosa* 1432 | 0 | 0 | 0 | 0 |
| *P. aeruginosa* 1437 | 0 | 0 | 0 | 0 |
| *P. aeruginosa* 1463 | 0 | 0 | 0 | 0 |
| *P. aeruginosa* 1479 | 0 | 0 | 0 | 0 |
| *P. aeruginosa ATCC* 27853 | 0 | 0 | 0 | 0 |

a) Shown are the number of recipients per 10$^8$ colony forming units (CFU) in two separate experiments. *P. oleovorans* and the listed *P. aeruginosa* strains were incubated together in the presence or absence of *A. castellanii* for 24 hours in 1% PYG in PBS (v/v), after which the contents were added to tryptic soy agar to determine CFU counts as well as to selective plates containing 4 mg/L meropenem, 2 mg/L tobramycin and 25 mg/L 1,10-phenanthroline to determine the number of recipients.

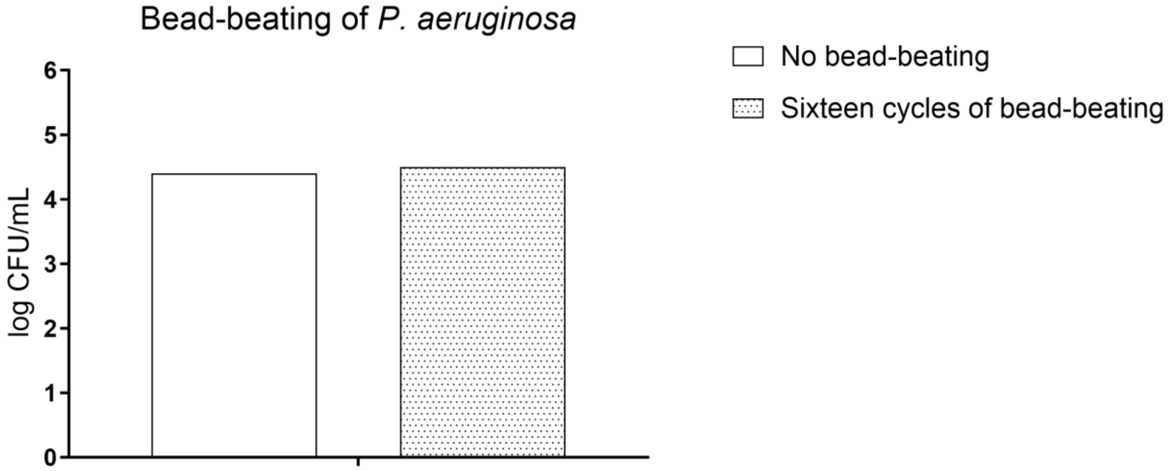


**Figure S1. Effect of sixteen cycles of bead-beating on *P. aeruginosa* colony-forming unit (CFU) count.** A suspension of *P. aeruginosa* was subjected to sixteen cycles (30 seconds on and 30 seconds off) of bead-beating with 1 mm glass beads using a Qiagen TissueLyser II. Before and after bead-beating, a sample was added to tryptic soy agar to determine CFU counts. This experiment was performed once.

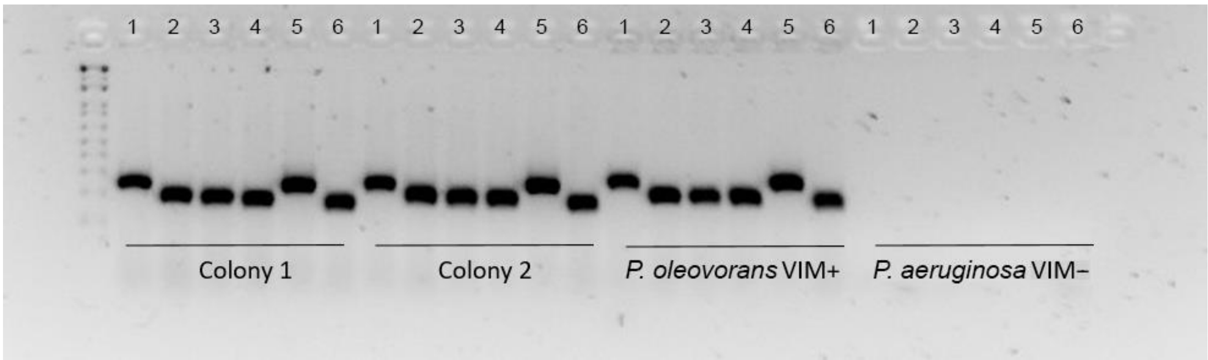


**Figure S2. Conventional PCR analysis to examine the intactness of the *bla*$_{VIM-2}$-containing plasmid in recipients.** *P. oleovorans* and *P. aeruginosa* were incubated in the presence or absence of *A. castellanii* for 24 hours, after which the cells were added to selective plates. A total of 56 colonies present on the selective plates were tested for the presence of the entire plasmid. Shown are the results of two colonies and the positive (*P. oleovorans*) and negative (*P. aeruginosa*) controls. The other 54 colonies showed the same positive result (not shown). The target genes (present on the *bla*$_{VIM-2}$-containing plasmid, see Figure S3) are indicated above the respective lanes. 1: *dotG*; 2: *pdeF*; 3: *chpB*; 4: *bla*; 5: *mobA*; 6: *pilP*.

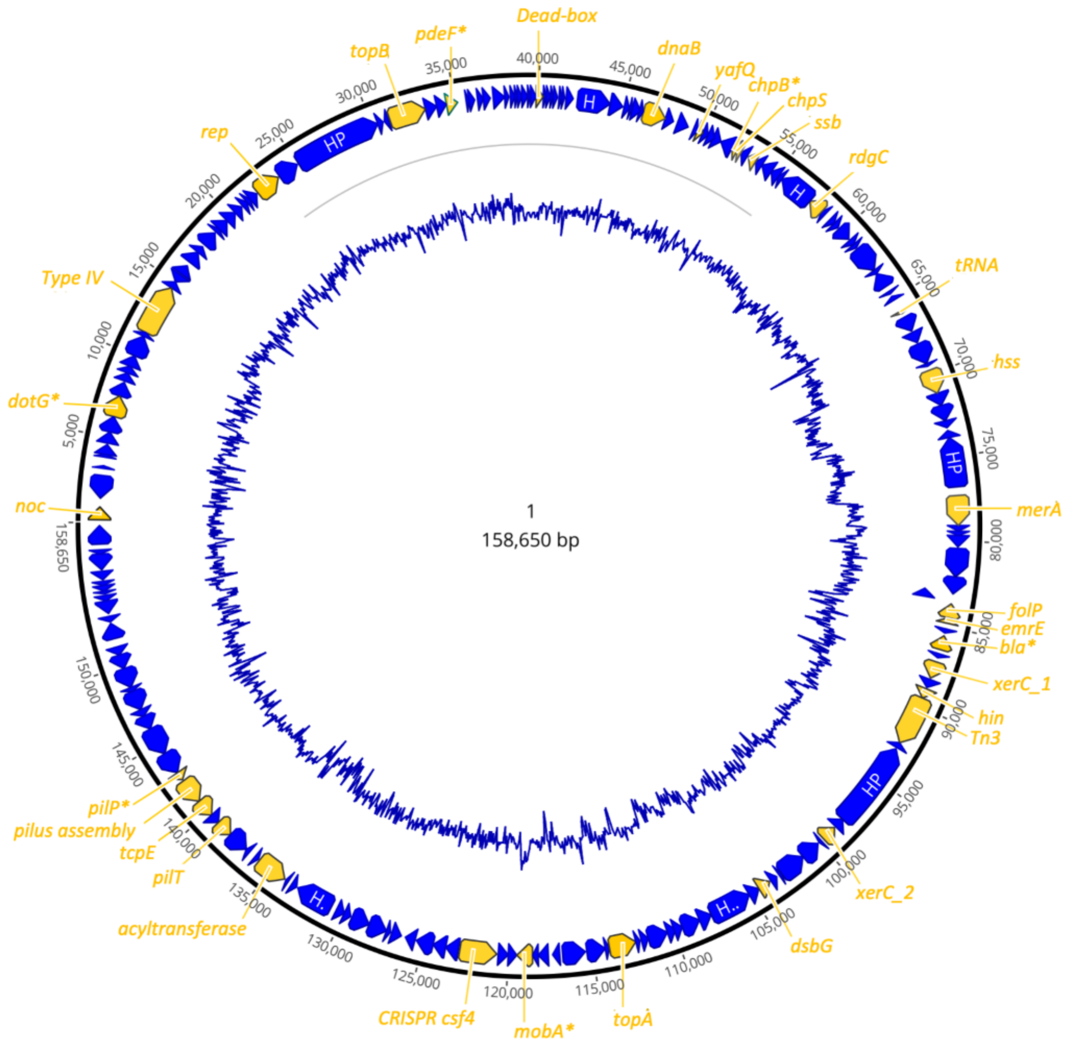


**Figure S3. Plasmid map of the *bla*$_{VIM-2}$ containing plasmid from *P. oleovorans*. The plasmid sequence is visualised in Geneious Prime (geneious, Auckland, New Zealand).** Annotated genes are indicated in yellow. The target genes used to determine the presence of the entire plasmid are indicated with an asterisk.
